# Response to Comment on: Does every transgender person want gender affirming surgery? A survey of transgender individuals in the Midwestern United States

**DOI:** 10.1038/s41443-025-01041-6

**Published:** 2025-03-25

**Authors:** Helen H. Sun, Braveheart Gillani, Shubham Gupta, Swagata Banik, Rachel Pope

**Affiliations:** 1 Urology Institute, University Hospitals Cleveland Health System, Cleveland, OH USA; 2 Case Western Reserve University School of Medicine, Cleveland, OH USA; 3 Deperro School of Health Professions at St. Bonaventure University, St. Bonaventure, NY USA

**Keywords:** Quality of life, Preclinical research

We appreciate the thoughtful commentary provided by Dr. Çeker on our study, “Does every transgender person want gender affirming surgery? A survey of transgender individuals in the Midwestern United States” [1, 2]. Dr. Çeker offers notable insights into key findings of our study and also highlights the limitations and challenges of examining transgender and gender diverse (TGD) individuals’ health. These perspectives have enriched our understanding of the complexities involved in researching TGD populations.

This study was undertaken with the desire to better understand motivations and barriers to access to gender-affirming surgery (GAS) for TGD populations within the Midwest. As Dr. Çeker notes, we acknowledge that our use of a convenience sample limits the generalizability of our findings to TGD individuals in different regions with varying ethnicities, healthcare structures, and societal expectations.

Our survey was developed in collaboration with a diverse group of stakeholders from the TGD community in addition to focus groups of TGD volunteers within hospital and community settings. This approach aimed to ensure the instrument was sensitive to the varied experiences during the transition process. Despite our efforts to capture the nuanced desires and preferences of binary and non-binary TGD individuals, we recognize the limitations of our targeted questions in fully representing these distinctions, as indicated by Dr. Çeker. Though 78% of participants reported having insurance for gender-affirming health care, this did not always translate into ease of insurance usage or coverage for GAS. Anecdotally, our patients have cited additional barriers such as transportation (sometimes across state borders) and hair removal (which is not covered by insurance), as costs and logistics which are prohibitive. While our respondents were vague about specific financial constraints, many cited hostile employment environments, highlighting upstream challenges at the systemic level.

Dr. Çeker also astutely points out that our study revealed knowledge gaps in both healthcare providers and TGD individuals seeking GAS. One-fifth of participants were not aware of the WPATH criteria, and 7% felt that their providers did not have specialized training in gender-affirming health care. While participants noted “vague and unclear requirements” by insurance companies for GAS coverage, patients and providers may be further confused by rapidly changing legislation on gender-affirming health care bans, as well as misinformation regarding the safety and efficacy of GAS [3]. These factors underscore the need for clear communication and ongoing education for both patients and healthcare providers.

This study identified non-binary individuals as a group which may be at a particular disadvantage within our current healthcare system, signaling a critical need for the system to reassess and address their specific needs. We hope that future investigation through larger samples and focused interviews will improve healthcare delivery to this population.

We are grateful for Dr. Çeker’s valuable contributions and look forward to incorporating his feedback in our ongoing and future research efforts.
